# The use of plasma aldosterone and urinary sodium to potassium ratio as translatable quantitative biomarkers of mineralocorticoid receptor antagonism

**DOI:** 10.1186/1479-5876-9-180

**Published:** 2011-10-21

**Authors:** Rena J Eudy, Vaishali Sahasrabudhe, Kevin Sweeney, Meera Tugnait, Amanda King-Ahmad, Kristen Near, Paula Loria, Mary Ellen Banker, David W Piotrowski, Carine M Boustany-Kari

**Affiliations:** 1 Department of Cardiovascular, Metabolic, and Endocrine Diseases, Pfizer, Eastern Point Road, Groton, CT, USA; 2 Department of Clinical Pharmacology, Pfizer, Eastern Point Road, Groton, CT, USA; 3 Department of Pharmacokinetics, Pharmacodynamics and Metabolism, Pfizer, Eastern Point Road, Groton, CT, USA; 4 Department of Pharmatherapeutics Research CoEs, Pfizer, Eastern Point Road, Groton, CT, USA; 5 Boehringer Ingelheim, Ridgefield, CT, USA

## Abstract

**Background:**

Accumulating evidence supports the role of the mineralocorticoid receptor (MR) in the pathogenesis of diabetic nephropathy. These findings have generated renewed interest in novel MR antagonists with improved selectivity against other nuclear hormone receptors and a potentially reduced risk of hyperkalemia. Characterization of novel MR antagonists warrants establishing translatable biomarkers of activity at the MR receptor. We assessed the translatability of urinary sodium to potassium ratio (Na^+^/K^+^) and plasma aldosterone as biomarkers of MR antagonism using eplerenone (Inspra^®^), a commercially available MR antagonist. Further we utilized these biomarkers to demonstrate antagonism of MR by PF-03882845, a novel compound.

**Methods:**

The effect of eplerenone and PF-03882845 on urinary Na^+^/K^+ ^and plasma aldosterone were characterized in Sprague-Dawley rats and spontaneously hypertensive rats (SHR). Additionally, the effect of eplerenone on these biomarkers was determined in healthy volunteers. Drug exposure-response data were modeled to evaluate the translatability of these biomarkers from rats to humans.

**Results:**

In Sprague-Dawley rats, eplerenone elicited a rapid effect on urinary Na^+^/K^+ ^yielding an EC_50 _that was within 5-fold of the functional *in vitro *IC_50_. More importantly, the effect of eplerenone on urinary Na^+^/K^+ ^in healthy volunteers yielded an EC_50 _that was within 2-fold of the EC_50 _generated in Sprague-Dawley rats. Similarly, the potency of PF-03882845 in elevating urinary Na^+^/K^+ ^in Sprague-Dawley rats was within 3-fold of its *in vitro *functional potency. The effect of MR antagonism on urinary Na^+^/K^+ ^was not sustained chronically; thus we studied the effect of the compounds on plasma aldosterone following chronic dosing in SHR. Modeling of drug exposure-response data for both eplerenone and PF-03882845 yielded EC_50 _values that were within 2-fold of that estimated from modeling of drug exposure with changes in urinary sodium and potassium excretion. Importantly, similar unbound concentrations of eplerenone in humans and SHR rats yielded the same magnitude of elevations in aldosterone, indicating a good translatability from rat to human.

**Conclusions:**

Urinary Na^+^/K^+ ^and plasma aldosterone appear to be translatable biomarkers of MR antagonism following administration of single or multiple doses of compound, respectively.

**Trial Registration:**

For clinical study reference EE3-96-02-004, this study was completed in 1996 and falls out scope for disclosure requirements.

Clinical study reference A6141115: http://clinicaltrials.gov, http://NIHclinicaltrails.gov; NCTID: NCT00990223

## Background

Accumulating evidence supports the role of the mineralocorticoid receptor (MR) in the pathogenesis of various diseases such as hypertension [1], diabetic nephropathy [2], and cardiac fibrosis [3]. Aldosterone, the primary endogenous ligand for MR, plays a pivotal role in the reabsorption of Na^+ ^and the excretion of K^+ ^in various epithelia such as the distal nephron [4] and the colon [5]. In addition to its essential role in the regulation of electrolyte balance, MR has been localized to numerous non-epithelial tissues where it has been implicated in the development of fibrosis, inflammation, and oxidative stress [6]. Current marketed MR antagonists, such as spironolactone and eplerenone, are mainly indicated for the treatment of hypertension and various forms of congestive heart failure. However, given the accumulating evidence supporting a role for MR antagonists in numerous additional diseases such as diabetic nephropathy, pharmaceutical companies have expressed a renewed interest in compounds that antagonize MR. Newer generations of MR antagonists are now being explored for novel indications.

To support the preclinical and clinical development of novel MR antagonists, translatable biomarkers are needed early in the discovery process to select compounds with the greatest likelihood of achieving the warranted efficacy in the target patient population. One advantage for this class of compounds is the availability of prior data generated with marketed MR antagonists, such as eplerenone and spironolactone, that can be leveraged to assess the translatability of biomarkers. The challenge remains to identify animal models in which the same biomarkers are equally robust. Animal models of disease often do not express all the traits of the human pathology. This is particularly true for animal models of diabetic nephropathy [7-9]. Additionally, most preclinical studies focus on renal histopathological changes to demonstrate drug effects thereby hindering translatability of their findings to humans [10,11]. Therefore, identifying and validating translatable mechanism biomarkers for drug interventions targeting this disease are essential.

Previous reports have indicated a measurable effect of MR antagonists on urinary sodium to potassium ratio (Na^+^/K^+^) in preclinical species, consistent with effects of aldosterone on electrolyte balance [12-14]. Moreover, elevations in plasma renin activity (PRA) and aldosterone were demonstrated in response to eplerenone treatment [15,16]. Despite these elevations in components of the renin angiotensin system, eplerenone decreased tubular Na^+ ^reabsorption, highlighting the importance of aldosterone in regulating electrolyte balance [15]. We provide herein evidence for the quantitative translatability from rats to humans of urinary Na^+^/K^+ ^as a mechanism biomarker for MR blockade following administration of single doses of compounds. Furthermore, we demonstrate a time- and dose-dependent increase in aldosterone with chronic MR blockade, and we further highlight the translatability of this biomarker to humans.

## Methods

### *In vitro *potency

Huh7 cells (ATCC, Manassas, VA) were transiently transfected with a luciferase reporter gene under the control of a Gal4 response element (Gal4-RE-luc) and a plasmid containing the Gal4 DNA binding domain fused to the MR ligand binding domain (Gal4-MR-LBD). Cells were treated with a submaximal level of ligand (~EC_80_) in the presence or absence of compounds, in a serum free media. Measurement of luciferase activity allowed for a quantitative determination of the reporter transcription in the presence of competitive antagonists, thus yielding an IC_50 _for these compounds.

### Effect of PF-03882845 and eplerenone on urinary sodium to potassium ratio (Na^+^/K^+^) in Sprague-Dawley rats

All procedures were conducted in accordance with Institutional Animal Care and Use Committee guidelines and regulations at Pfizer Inc. (Groton, CT). Male, carotid artery catheterized Sprague-Dawley rats were received from Charles River laboratories at approximately 11 weeks of age (body weight = 300-400 g). Rats were singly housed in wire cages on a 12-hour light cycle and were provided standard laboratory chow diet and water *ad libitum *prior to and throughout the studies. Following acclimation, animals were randomly assigned to treatment groups (n = 6/group). In one experiment, rats received single oral doses of 3, 10, or 30 mg/kg PF-03882845 or vehicle (0.5% methylcellulose and 0.1% polysorbate 80) in a dosing volume of 5 mL/kg. In a second experiment, rats received single oral doses of 5, 30, or 300 mg/kg eplerenone or vehicle (0.5% methylcellulose and 0.1% polysorbate 80) in a dosing volume of 5 mL/kg. Blood samples were collected via catheter at 1, 2, 4 and 7 hours post-dose for measurement of plasma drug exposure. Urine samples were collected overnight prior to dosing and at intervals of 0-2, 2-4, and 4-7 hours post-dose for measurement of urinary sodium and potassium concentration.

### Temporal effect of PF-03882845 on plasma aldosterone in Spontaneously Hypertensive Rats (SHR)

To determine the time course for aldosterone modulation by MR antagonists, male carotid artery catheterized SHR (11 weeks of age, body weight = 250-300 g, from Charles River laboratories, Portage, MI) were randomly assigned (n = 9/group) to receive 30 mg/kg PF-03882845 administered BID by oral gavage (first dose administered at 6AM, second dose administered at 4PM) or vehicle (0.5% methylcellulose and 0.1% polysorbate 80) for a duration of 1, 3, 5 or 7 days. Throughout the study, rats were provided standard laboratory chow diet and water *ad libitum*. On days 1, 3, 5 and 7, blood samples were collected via catheter prior to dosing and at 1, 2, 4 and 7 hours post-dose for measurement of plasma aldosterone and drug levels. A different subset of SHR was used for each day of the time course, in order not to exceed the maximal allowed daily blood collection. The rationale for utilizing the SHR strain was to maintain consistency with additional undisclosed studies conducted in parallel with the above experiments. SHR, like Sprague-Dawley rats, possess a functional renin-angiotensin-aldosterone system (RAAS) [17,18].

### Effect of PF-03882845 and eplerenone on plasma aldosterone levels in SHR

To characterize the dose-response of PF-03882845 and eplerenone on plasma aldosterone levels, male carotid artery catheterized SHR (11 weeks of age, body weight = 250-300 g, from Charles River laboratories, Portage, MI) were randomly assigned (n = 9/group) to receive 20, 30 or 50 mg/kg PF-03882845 twice daily (BID) by oral gavage for 5 days, or 50, 150, or 450 mg/kg eplerenone BID by oral gavage for 7 days. The control group was treated with vehicle (0.5% methylcellulose and 0.1% polysorbate 80). Throughout the study, rats were provided standard laboratory chow diet and water *ad libitum*. On the final study day, blood and serum samples were collected via catheter prior to dosing (6AM) and at 1, 2, 4 and 7 hours post-dose for measurement of aldosterone and drug levels.

### Pharmacodynamic measurements in rats

Urinary sodium and potassium concentrations were measured using a Siemens Advia 1800 chemistry analyzer. Plasma aldosterone levels in rats were measured using a RIA kit from Diagnostic Systems Laboratories (Webster, TX) with a lower limit of quantitation (LLOQ) of 25 pg/mL. Concentrations of aldosterone in human serum were determined using a validated LC/MS/MS method with a LLOQ of 1 ng/dL (Quest Diagnostics).

### Determination of PF-03882845 and eplerenone in rat plasma

Mass spectrometry was performed on a Sciex API4000 system equipped with a turbo-ionspray source (Applied Biosystems, Foster City, CA, USA) operated in negative ion mode for PF-03882845 and in positive ion mode for eplerenone. High performance liquid chromatography (HPLC) analysis was conducted on a Shimadzu 10ADvp Binary HPLC system (Shimadzu Scientific Instruments Columbia, MD, USA) with a CTC-PAL (Thermo Scientific, Franklin, MA, USA) as the autosampler. Chromatographic separations were performed by reversed-phase gradient elution of each compound and the internal standard on a Phenomenex Luna C18 (2) 30 × 2 mm 5 μ HPLC column (Torrance, CA, USA) using 10 mM ammonium acetate and 1% isopropyl alcohol in water and acetonitrile. Analyst (version 1.4.1, Applied Biosystems, Foster City, CA, USA) was employed to control the instrument operation and acquire data in multiple reaction monitoring (MRM) mode. The ion transitions for PF-03882845 and the internal standard were 418.2 → 374.1 and 356.1 → 312.1, respectively. The dynamic range of the assay was from 1 ng/mL to 10,000 ng/mL using linear regression with a weighting of 1/x^2^. The ion transitions for eplerenone and the internal standard were 415 → 163.5 and 368.1 → 112.1, respectively. The dynamic range of the assay was from 1 ng/mL to 5,000 ng/mL, using linear regression with a weighting of 1/x^2^. Unbound plasma drug concentrations for PF-03882845 and eplerenone were determined by multiplying total plasma concentration by the fraction of drug unbound in rat plasma (fu = 0.0038 for PF-03882845, and fu = 0.81 for eplerenone).

### Non clinical data analysis

The relationship between urinary log10 (Na^+^/K^+^) data and plasma concentrations of eplerenone or PF-03882845 was described using an indirect response model depicted in the clinical data analysis section. Data analysis for plasma aldosterone was performed using Graphpad Prism 5. EC_50 _values for each compound were determined by fitting the log of the average drug concentration over 0 to 7 hours (determined by area under the curve (AUC)_0-7_/7) versus the aldosterone AUC_0-7 _to a simple Emax model. The free drug concentration required to elicit a 2-fold increase in aldosterone was determined using the interpolation function for the log (drug concentration) versus response curve fit. One-way ANOVA followed by Tukey's post- hoc analysis was used to establish significance between the treatment groups.

### Clinical Studies

A single center, double-blind, randomized, placebo-controlled, rising oral dose, sequential panel study was conducted in 32 healthy male subjects (Study EE3-96-02-004). Subjects had a mean age of 33.4 years (range 19 to 62 years) and a mean body weight of 75.1 kg (range 61.4 to 91.0 kg). Of the 32 enrolled subjects, 31 were Caucasian and 1 was Asian. Subjects were in good general health as determined by the Investigator in the pre-treatment period. Subjects were excluded if they had any history of renal, hepatic, cardiovascular, gastrointestinal, or endocrinologic abnormality. Every subject provided written informed consent prior to admission to the study. Subjects were administered 100, 300 or 1000 mg of eplerenone or placebo on day 1, followed 48 hours later by the same dose administered once-a-day for 11 consecutive days. On day 1 and after 11 days of dosing, blood samples were drawn at 0 (pre-dose) and 0.5, 1, 2, 3, 4, 6, 8, 12, 16 and 24 hours post-dose for determination of plasma drug concentration. Urine samples were collected at 0-2, 2-4, 4-6, 6-8, 8-12, and 12-24 hours post-dose on days 1 and 11 for determination of electrolyte levels. A pre-dose 24 hour urine sample was also collected from each subject on day 0. Subjects were maintained on an in-house controlled salt diet from three days prior to the first dose administration until day 14. The controlled salt diet restricted subjects to a daily intake of 150 mmol of sodium and 80 mmol of potassium.

A methodology study to evaluate the pharmacodynamic (PD) response of a 100 mg oral dose of eplerenone administered once daily for 10 days to healthy volunteers was also conducted (Study A6141115). Twenty (20) subjects (age 18 - 55 years) in the single cohort were randomized as 15 on eplerenone and 5 on placebo. All subjects enrolled in this study were male with a mean age of 39.5 years in the eplerenone group and 36.4 years in the placebo group. The mean body weight of the subjects was 79.4 kg (range 70.0 to 101.8 kg) in the eplerenone group and 80.3 kg (range 76.4 to 86.8 kg) in the placebo group. Of the 20 enrolled subjects, 18 were Caucasian and 2 were African-American. Generally, demographic characteristics were comparable between treatment groups. Subjects were in good general health as determined by the Investigator in the pre-treatment period. Subjects were excluded if they had any history of renal, hepatic, cardiovascular, gastrointestinal, or endocrinologic abnormality. Every subject provided written informed consent prior to admission to the study. Subjects were maintained on an in-house controlled salt diet from three days prior to the first dose of study drug until day 13 of the study. Dosing was for 10 consecutive days; beginning on day 1, and subjects received once daily oral doses of 100 mg eplerenone or placebo. Serial blood samples were collected from each subject on days 1 and 10 for measurement of plasma eplerenone levels. Serum aldosterone (PD biomarker) levels were measured on days 0, 1 and 10 of the study.

### Determination of eplerenone concentration in human plasma

Plasma concentrations of eplerenone in study EE3-96-02-004 were determined using a validated LC/MS/MS method (Phoenix International Life Sciences, Montreal, Canada) with a LLOQ of 10 ng/mL. Plasma concentrations of eplerenone in study A6141115 were determined using a validated LC-MS/MS method with a LLOQ of 10 ng/mL (WuXi AppTec, Shanghai, China).

### Clinical data analysis

Urinary log10 (Na^+^/K^+^) was analyzed based on measurements obtained following a single dose (day 1) and multiple doses (day 11) of study drug. An indirect response model was used to describe the relationship between the urinary Na^+^/K^+ ^and plasma concentrations of eplerenone on each day:

$$\begin{array}{c} \frac{dR}{dt}=k_{in}*\left(1+STIM\right)-k_{out}*R\\ STIM=PBO+\frac{E\max^{*}Conc}{EC_{50}+Conc} \end{array}$$

where R = urinary Na^+^/K^+^, kin = appearance rate constant, kout = removal rate constant, STIM = drug effect, Conc = average plasma concentration of eplerenone during the collection interval, PBO = placebo response, Emax = maximal eplerenone stimulatory effect and EC_50 _= concentration producing 50% half-maximal stimulation. Modeling was performed using nonlinear mixed effects modeling methodology as implemented in the NONMEM software system, version VI level 1.2 (GloboMax LLC, Hanover, MD).

To evaluate the aldosterone (PD biomarker) response to 100 mg eplerenone, the area under the effect curve from time 0 to 24 hours area under the effective curve (AUEC [0 24]), was calculated for serum aldosterone for each subject and day using the linear trapezoidal method. Baseline values were defined as the AUEC values on day 0. In addition, the linear mixed effect repeated measures models were used to analyze the natural log transformed AUEC with treatment, day and treatment by day interaction as fixed effects, natural log transformed AUEC day 0 value as covariate, and subject as a random effect. The adjusted mean differences and 90% confidence intervals (CI) for the differences were obtained from the model and exponentiated to provide estimates of the ratio of adjusted geometric means and 90% CI for the ratios of each treatment.

## Results

### Functional potency of mineralocorticoid receptor antagonists

PF-03882845 potently antagonized MR with a geometric mean IC_50 _of 0.75 nM (90% CI: 0.504-1.11; n = 6) in Huh7 cells transiently transfected with MR-LBD, in a serum free media. Similarly, eplerenone antagonized MR with an IC_50 _of 109 nM (90% CI: 79.3-150; n = 6).

### Effect of MR antagonists on urinary Na^+^/K^+^

**Figure 1:**
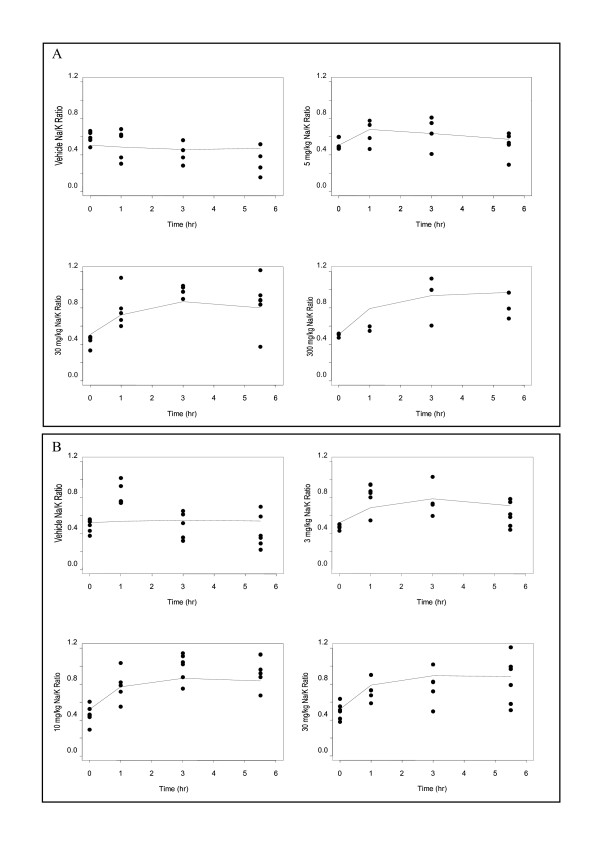
**Effect of eplerenone and PF-03882845 on urinary Na^+^/K^+ ^in rats**. Eplerenone (A) and PF-03882845 (B) elicited a dose-dependent increase in urinary Na^+^/K^+ ^ratio in Sprague-Dawley rats following administration of single doses. Points represent observed effects in individual rats. The solid line represents the predicted time course of the effect derived from the indirect response model described in the methods section.

To determine the effects of MR antagonists on electrolytes, urinary Na^+^/K^+ ^was measured following a single oral administration of 3, 10 or 30 mg/kg PF-03882845 or 5, 30 or 100 mg/kg eplerenone to Sprague-Dawley rats. Both eplerenone and PF-03882845 resulted in an increase in urinary Na^+^/K^+ ^over 7 hours that was concentration-dependent (Figure 1). Longitudinal models incorporating no drug effect (placebo/vehicle), linear drug effect or an Emax drug effect were evaluated for eplerenone and/or PF-03882845 preclinical and clinical data. Model selection was performed based on standard diagnostic plots and the comparison of objective function values using the likelihood-ratio test [19,20]. The longitudinal model with an Emax drug effect when compared to a model with no drug effect resulted in a p-value < 0.001 indicating the superiority of a concentration-dependent Emax model for describing the data. Diagnostic plots supported the likelihood ratio test for model selection.

Consistent with differences in *in vitro *potency between the two compounds, the estimated free EC_50 _of eplerenone and PF-03882845 from the indirect response model were 460 nM and 1.90 nM, respectively. When eplerenone was administered for a duration of 7 days, effects on urinary Na^+^/K^+ ^were no longer apparent. Similarly, effects on urinary Na^+^/K^+ ^following 3 days of dosing with PF-03882845 were attenuated compared to effects observed after a single dose (data not shown).

**Figure 2:**
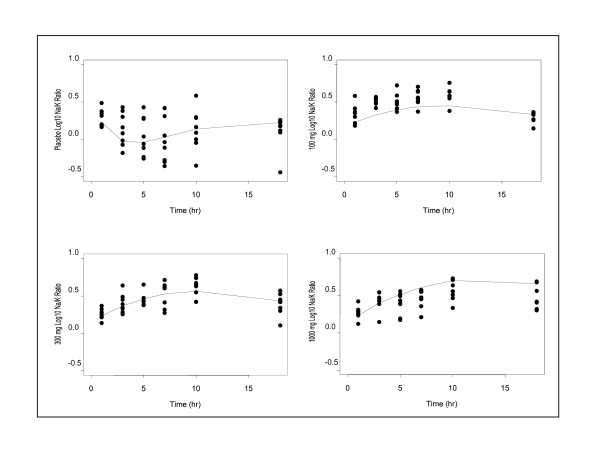
**Effects of eplerenone on urinary log10 Na^+^/K^+ ^in humans**. Eplerenone elicited a dose-dependent increase in urinary Na+/K+ in humans. Points represent observed effect in individual subjects. The solid line represents the predicted time course of the effect derived from the indirect response model described in the methods section.

In humans, eplerenone elicited an increase in urinary Na^+^/K^+ ^ratio following administration of single oral doses of 100, 300 or 1000 mg (Figure 2). In this study, eplerenone was well tolerated during single- and multiple-dose administration. There were no clinically significant changes in physical examinations, vital signs (including blood pressure), ECG, or clinical laboratory tests (including serum potassium, sodium levels and renal function). Consistent with preclinical findings, effects of eplerenone in healthy humans were concentration-dependent. The estimated EC_50 _for the effect of eplerenone on urinary Na^+^/K^+ ^was 557 nM, demonstrating a strong translatability from rats to humans. Moreover, the effect of eplerenone on urinary Na^+^/K^+ ^ratio was not sustained with chronic dosing (data not shown). The effect of PF-03882845 on urinary Na^+^/K^+ ^has not yet been studied in humans.

### Effect of MR antagonists on plasma aldosterone

**Figure 3:**
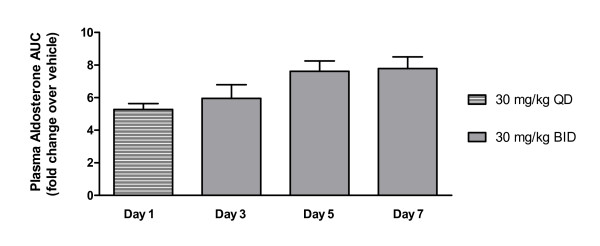
**Time-dependent effects of PF-03882845 on plasma aldosterone AUC**. PF-03882845 resulted in a time-dependent increase in plasma aldosterone AUC corrected to vehicle in Spontaneously Hypertensive Rats (SHR). Statistical analysis indicated an overall significant effect of time (P = 0.0454 by one-way ANOVA). There was no significance between any two groups when analyzed with Tukey's Multiple Comparison Test. Data are shown as mean + SEM.

To characterize the time-course of MR antagonism on aldosterone changes, plasma aldosterone levels were measured following a single (1 day) and multiple administration (BID for 3, 5 or 7 days) of 30 mg/kg PF-03882845 to SHR. Results indicated a time-dependent increase in aldosterone levels up to 5 days of BID dosing (Figure 3). A maximal effect was reached by day 5 for this compound, as day 7 levels did not differ from day 5 despite a continued increase of the drug concentration (average concentration on day 5 was 23.2 nM, average concentration on day 7 was 67.0 nM). These results indicate that chronic dosing is warranted in order to achieve a maximal effect on aldosterone.

**Figure 4:**
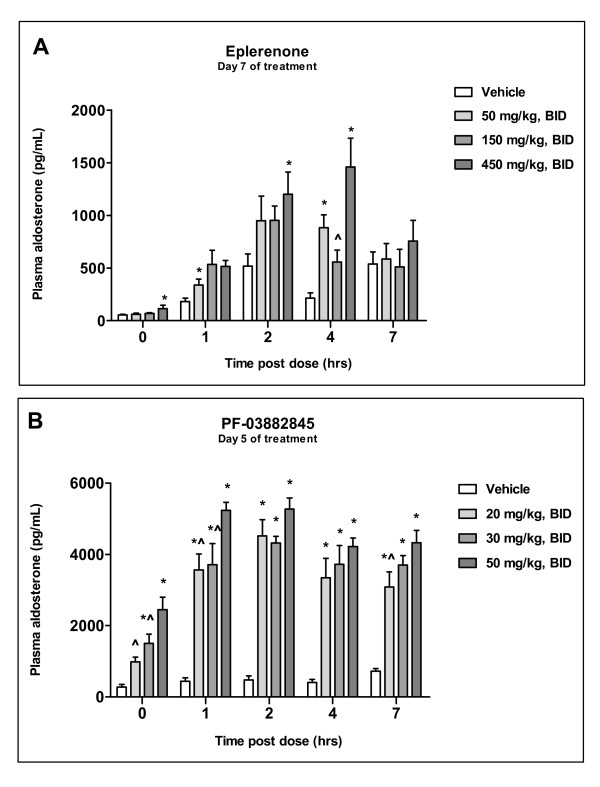
**Effect of chronic administration of eplerenone and PF-03882845 on plasma aldosterone**. Treatment of Spontaneously Hypertensive Rats (SHR) with eplerenone for 7 days caused significant increases in plasma aldosterone (A) yielding a calculated EC_50 _of 764 nM. Treatment with PF-03882845 for 5 days resulted in significant elevations in aldosterone (B) and yielded an EC_50 _of 3.08 nM. All data are represented as mean + SEM. In Figure A,* and ^ indicate a significant difference from vehicle and 450 mg/kg BID, respectively. In Figure B, * and ^ indicate a significant difference from vehicle and 50 mg/kg BID, respectively.

To characterize the dose-response of eplerenone and PF-03882845 on plasma aldosterone levels, SHR were treated with either PF-03882845 at doses of 20, 30 and 50 mg/kg for 5 days, or eplerenone at doses of 50, 150, or 450 mg/kg for 7 days, or vehicle (0.5% methylcellulose and 0.1% polysorbate 80). Treatment with eplerenone resulted in a dose-responsive increase in aldosterone with an EC_50 _of 764 nM (Figure 4A). PF-03882845 elicited an increase in aldosterone that was sustained over 7 hours post-dose, with an EC_50 _of 3.08 nM (Figure 4B). These potencies are within 2-fold of that determined from the concentration effect curves on urinary Na^+^/K^+ ^ratio.

The effect of eplerenone on serum aldosterone in humans was assessed at a dose of 100 mg administered once daily for 10 consecutive days in healthy volunteers. In this study, treatment with eplerenone was safe and well-tolerated. There were no clinically significant changes from baseline for any hematology, clinical chemistry (including serum potassium or sodium levels), or urinalysis test values. In addition, there were no changes in vital sign parameters from baseline (including blood pressure) that were assessed as clinically significant. Following 10 days of eplerenone or placebo administration, serum aldosterone AUC was calculated and normalized to serum aldosterone AUC at baseline (day 0). The dose of 100 mg eplerenone resulted in a 2.2-fold increase in aldosterone day 10 AUC/day 0 AUC versus placebo with an average unbound plasma drug concentration of 491 nM, as measured on day 10 of dosing. In comparison, an average eplerenone unbound plasma concentration of 514 nM in rats resulted in a 2.2-fold increase in aldosterone versus vehicle, when eplerenone was administered for 7 days. These results indicate a high translatability for aldosterone modulation by MR antagonists from rat to human.

## Discussion

We studied the effect of MR blockade on urinary Na^+^/K^+ ^and aldosterone. Our findings demonstrate the translatability of urinary Na^+^/K^+ ^from rat to human as a mechanism biomarker for acute effects of MR antagonism. Moreover our results indicate that aldosterone is a translatable mechanism biomarker following chronic administration of MR antagonists. These findings are of crucial importance for the clinical development of novel MR antagonists.

In the distal nephron, aldosterone, through effects on MR, regulates sodium balance by increasing the expression of the epithelial Na^+ ^channel (ENaC) [21] and the Na^+^/K^+^-ATPase pump found respectively on the apical and the basolateral membrane of the distal epithelial cells. These increases are at least partially driven through effects on the sodium/glucocorticoid kinase 1 (SGK1) which has been shown to be strongly induced by aldosterone. In turn, SGK1 has been demonstrated to phosphorylate the ubiquitin-ligase Nedd4-2 [22,23], thereby impairing its ability to degrade ENaC. The net effect is an increase in ENaC pool at the apical membrane. In accordance with the crucial role of aldosterone in salt reabsorption, MR blockade elicited an increase in urinary Na^+^/K^+ ^in rats and humans, in a dose-dependent manner. Importantly, the potency of eplerenone in this assay was highly translatable from rat to human, supporting the use of urinary Na^+^/K^+ ^in rats to enable dose selection for clinical trials.

Interestingly, increases in urinary Na^+^/K^+ ^were not sustained with chronic dosing in rats treated with eplerenone or PF-03882845. Although the reason for this loss of effect is not clearly understood, potential compensatory mechanisms could account for this finding. As shown in our study and in previous reports [15,16,24,25], MR antagonism is associated with a feedback increase in PRA (see Additional File 1), and therefore angiotensin II (AngII). The latter has been demonstrated to exert direct effects through the angiotensin type 1 (AT1) receptor on sodium channels [26]. Indeed, Beutler et al. revealed a decrease in α-ENaC protein and mRNA abundance in response to AT1 receptor blockade in Na^+^Cl^- ^restricted rats [27]. Importantly, this effect was not altered by spironolactone administration suggesting a direct regulation of α-ENaC by AngII, independent of aldosterone and MR. Other regulators of salt and water balance include vasopressin [28] which has been shown to result in an increase in α-, β- and γ-ENaC subunits in the rat renal collecting duct with chronic infusion [29,30]. This effect appears to be largely driven through translational regulations. Glucocorticoids have also been shown to stimulate renal sodium reabsorption through effects on several transporters, namely the sodium-hydrogen exchange by Na^+^/H^+ ^exchanger 3 in the proximal tubule [31], the Na-K-Cl cotransporter (NKCC 2) in the thick limb of Henle [32], the Na-Cl cotransporter (NCC) in the distal tubule [32,33], and ENaC in the connecting tubule and collecting ducts [34]. Indeed, early on, Stanton demonstrated that chronic infusion with dexamethasone in adrenalectomized rats increased sodium reabsorption, albeit to a lesser extent than physiological levels of aldosterone [35]. Thus, it is possible that compensatory increases in either glucocorticoids or vasopressin may offset the chronic effect of MR antagonists on urinary Na^+^/K^+^. However, in the absence of evidence supporting the above hypotheses, our data only support a role for PRA and therefore AngII in eliciting these compensatory responses but does not preclude a role for additional culprits such as vasopressin or glucocorticoids. Since clinical and preclinical data indicate a loss of effect on urinary Na^+^/K^+ ^following chronic treatment with eplerenone; thereby precluding the use of this biomarker in chronic studies, we investigated changes in aldosterone in rats and humans as a mechanism biomarker of chronic MR antagonism.

In the present experiments, MR antagonists induced an increase in serum aldosterone levels. These findings are concordant with previous reports. Menard et al, reported an increase in plasma aldosterone after two weeks of spironolactone treatment in SHR on high salt-normal potassium diet [36]. This has been confirmed in normal rats treated chronically with eplerenone [37]. Likewise, De Paula, et al reported an increase in both PRA and aldosterone after eplerenone treatment for 10 days in lean dogs [15]. Similarly, in humans, eplerenone resulted in elevations in serum aldosterone (65%) and plasma active renin (94%) when administered for 8-weeks in addition to a fixed-dose of an angiotensin converting enzyme (ACE) inhibitor or angiotensin receptor blocker (ARB) in mildly hypertensive patients [16]. In a larger study, Weinberger et al. reported an increase in serum aldosterone with eplerenone treatment that was dose-responsive [38]. In patients with resistant hypertension, Calhoun et al. showed an inverse correlation between blood pressure and serum aldosterone after treatment with eplerenone for 12 weeks [39]. We expand on these findings by demonstrating translatability of this biomarker from rat to human.

Further, we demonstrate a time-dependent increase in aldosterone with MR antagonism, as maximum levels of circulating aldosterone were not reached until day 5 with PF-03882845 despite an increase in drug exposure levels. This delay in achieving the maximal response is likely a result of a feedback mechanism to offset the loss of MR-mediated aldosterone effects. AngII and K^+ ^have been shown to be the main physiological regulators of aldosterone synthase [40], increasing mRNA levels of the enzyme in a time and concentration dependent manner [41]. Thus, increases in PRA and AngII ensuing from a decreased Na^+ ^reabsorption in the proximal tubules, would lead to an up-regulation of aldosterone synthase, and consequently increases in aldosterone. Mechanisms independent of AngII may also be driving the rise in aldosterone. This is supported by the absence of correlation between aldosterone and AngII levels in congestive heart failure patients treated with an ACE inhibitor, in which elevations in aldosterone were not necessarily accompanied by changes in AngII [42]. Another potential explanation for the increase in aldosterone is a direct regulation of aldosterone synthase by MR. However this seems unlikely given the absence of a mineralocorticoid/glucocorticoid response element on the gene encoding for this enzyme in humans without familial hyperaldosteroneism-1 [43]. Nonetheless, the presence of MR in the adrenals [44] could lead to an indirect regulation of aldosterone synthase expression. Further studies are warranted to fully understand the mechanisms underlying the rise in aldosterone following MR antagonism.

## Conclusions

Urinary Na^+^/K^+ ^measured in rats constitutes a valid quantitative mechanism biomarker of MR antagonism, as it correlates with the *in vitro *IC_50 _of the compounds tested. Importantly, this biomarker appears to be translatable to humans, as evidenced by clinical data generated with eplerenone. Aldosterone measurements in rats following treatment with MR antagonists yielded similar potencies to those calculated from effects on urinary Na^+^/K^+^. Furthermore, the same magnitude of aldosterone elevations were observed in rats and humans at similar unbound concentrations of eplerenone, thereby indicating translatability of this biomarker from rat to human. Taken together, our findings support the use of urinary Na^+^/K^+ ^as a translatable biomarker of MR antagonism in single dose studies. Moreover, our results support the use of aldosterone as a translatable biomarker of chronic MR blockade.

## List of Abbreviations Used

ACEi: angiotensin converting enzyme inhibitor; Ang II: angiotensin II; ARB: angiotensin receptor blocker; AT1: angiotensin I; AUC: area under the curve; AUEC: area under the effective curve; CI: confidence intervals; ENaC: epithelial Na^+ ^channel; HPLC: high performance liquid chromatography; LBD: ligand binding domain; LLOQ: lower limit of quantitation; MR: mineralocorticoid receptor; NKCC2: Na-K-Cl cotransporter^; ^NCC: Na-Cl cotransporter; PD: pharmacodynamic; PK: pharmacokinetic; PRA: plasma renin activity; RAAS: renin-angiotensin-aldosterone system; RIA: radioimmunoassay; SGK1: sodium/glucocorticoid kinase 1; SHR: spontaneously hypertensive rat.

## Competing interests

All authors were employees of Pfizer at the time this work was performed.

## Authors' contributions

RE carried out the *in vivo *portion of the non clinical studies (dosing, bleeding ect.), analyzed the plasma aldosterone data and co-drafted the manuscript. VS and KS performed the PK/PD modeling for the non clinical and clinical data and drafted the relevant sections of the manuscripts. MT contributed to the design of the non clinical studies and processed the PK data. AK performed the measurement of eplerenone and PF-03882845 plasma concentrations in non clinical samples and co-authored the relevant methods section. KN participated in the execution of the *in vivo *portion of the non clinical studies and the data analysis. PL and MEB designed and performed the *in vitro *experiments and co-authored the relevant methods sections. DWP participated in the design of the experiments. CMB conceived of the studies, oversaw their design and execution, and drafted this manuscript. All authors read and approved the final manuscript.

## Supplementary Material

Additional file 1 **Effect of chronic administration of PF-03882845 on plasma renin activity**. Treatment of Spontaneously Hypertensive Rats (SHR) with PF-03882845 for 7 days caused significant increases in plasma renin activity (PRA) at the dose of 50 mg/kg BID. * and ^ indicate significantly different from vehicle and 50 mg/kg BID, respectively. Data are depicted as mean + SEM (n = 9). Statistical analysis was performed using ANOVA followed by Tukey's post-hoc test. Click here for file
