# Supplementary figures and images for: The use of plasma aldosterone and urinary sodium to potassium ratio as translatable quantitative biomarkers of mineralocorticoid receptor antagonism
Source: J Transl Med. 2011 Oct 21;9:180. doi: 10.1186/1479-5876-9-180 (PMC3305907; doi:10.1186/1479-5876-9-180)

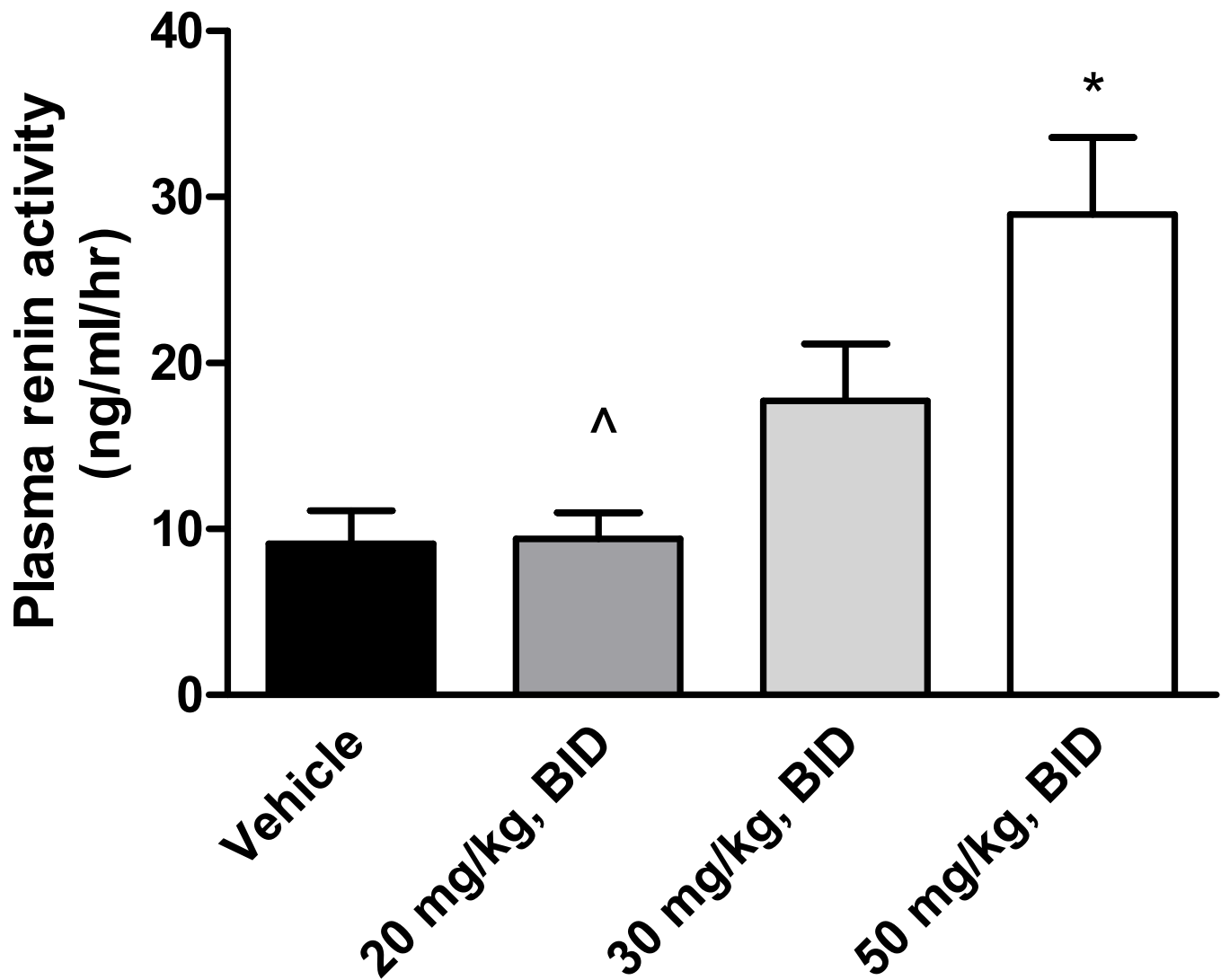

Supplement: Additional file 1 — Effect of chronic administration of PF-03882845 on plasma renin activity. Treatment of Spontaneously Hypertensive Rats (SHR) with PF-03882845 for 7 days caused significant increases in plasma renin activity (PRA) at the dose of 50 mg/kg BID. * and ^ indicate significantly different from vehicle and 50 mg/kg BID, respectively. Data are depicted as mean + SEM (n = 9). Statistical analysis was performed using ANOVA followed by Tukey's post-hoc test. [file 1479-5876-9-180-S1.PDF]
